# The Society for Women in Radiation Oncology Medical Physicists: Where Are We Five Years Later?

**DOI:** 10.7759/cureus.114277

**Published:** 2026-08-10

**Authors:** Raksha M Narasimhan, Morgan S Levy, Madison Blanco, Isabella B Dreyfuss, Rebecca F Krc, Taylor J Corriher, Maryam Mashayekhi, Sara B Ponce, Katie Lichter, Anna I Caldwell, Reshma Jagsi, Jenna M Kahn, Winnifred Wong, Adrianna Masters, Afua A Yorke, Crystal Seldon Taswell

**Affiliations:** 1 Department of Medical Education, University of Miami Miller School of Medicine, Miami, USA; 2 Department of Radiation Oncology, Markey Cancer Center, University of Kentucky Medical Center, Lexington, USA; 3 Department of Medical Education, Washington State University Elson S. Floyd College of Medicine, Spokane, USA; 4 Department of Radiation Oncology, University of Miami Miller School of Medicine, Miami, USA; 5 Department of Radiation Oncology, West Virginia University Medicine, Morgantown, USA; 6 Department of Radiation Oncology, Atrium Health Levine Cancer Institute, Charlotte, USA; 7 Department of Radiation Oncology, Mayo Clinic, Rochester, USA; 8 Department of Radiation Oncology, Radiation Oncology Associates, Ltd., Appleton, USA; 9 Department of Radiation Oncology, University of California San Francisco, San Francisco, USA; 10 Department of Radiation Oncology, Virginia Commonwealth University School of Medicine, Richmond, USA; 11 Department of Radiation Oncology, Winship Cancer Institute, Emory University, Decatur, USA; 12 Department of Radiation Oncology, Kaiser Northwest Permanente, Portland, USA; 13 Department of Radiation Oncology, Celilo Cancer Center, Adventist Health Columbia Gorge, The Dalles, USA; 14 Department of Radiation Oncology, University of Louisville School of Medicine, Louisville, USA; 15 Department of Radiation Oncology, University of Washington and Fred Hutch Cancer Center, Seattle, USA

**Keywords:** academic productivity, gender bias, gender equality, gender equity in radiation oncology, mentorship, physicist perspectives, swro survey, women physicists, workforce experiences, workplace barriers

## Abstract

Background

A better understanding of the personal experiences of women and gender minorities in medical physics is needed to ensure the vitality of the radiation oncology (RO) workforce. Founded in 2017, the Society for Women in Radiation Oncology (SWRO) sought to promote gender equity in RO and includes both physician and medical physicist members.

Methods

From January to February 2023, an anonymous 82-question survey was conducted among all current SWRO members, including both physicians and medical physicists, via the SWRO email listserv. Survey questions covered demographics, family planning, mentorship, well-being, perceptions of SWRO membership, and perceptions of the RO field. A sub-analysis was performed on the survey responses from medical physicists. Descriptive statistics were utilized to summarize the findings.

Results

In this sub-analysis of physicists, 20 of 26 physicist members (77% participation rate), including 15 medical physicists and five resident physicists, completed the survey. The majority of survey respondents were in the 31-35 year old age group (35%) and female (95%). The top three limitations of academic productivity reported included clinical responsibilities (34%), insufficient mentorship (26%), and insufficient funding (16%). Twenty-eight percent of respondents reported feeling somewhat or extremely satisfied with the mentorship available at their institution, while 60% reported being somewhat or extremely satisfied with female mentorship in the field. Thirty-nine percent of respondents perceived gender-specific biases or obstacles within their program.

Among respondents, 44% reported unwanted sexual comments, attention, or advances by colleagues or superiors. Suggested areas for improvement included increasing education on career development, promoting greater physics representation within SWRO, enhancing integration of physics and RO events, and increasing opportunities for in-person interaction.

Conclusion

This sub-analysis of a broader SWRO survey presents the perspectives of women and gender minority physicists within RO, highlighting shared opportunities for improvement between RO physicians and physicists regarding female mentorship and gender-based challenges. This supports the importance of organizations such as SWRO in promoting representation and gender equity in RO.

## Introduction

Women have consistently been an underrepresented group in the fields of radiation oncology (RO) and medical physics. In 2020, women made up only 27.5% of the RO physician workforce [1]. Factors contributing to this lack of growth include, but are not limited to, a dearth of female role models, discrimination, and family planning [1,2]. In 2017-2018, survey results highlighted the experiences of female medical residents in RO [2]. At that time, more than one-quarter (29%) were in programs with ≤2 female residents and ≤2 female attendings (29%). Over half (51%) reported that a lack of mentorship affected their career ambitions, and 52% reported that gender-specific bias existed in their programs. Almost all (90%) participants in this study expressed interest in joining a professional organization for women in RO, supporting the continued expansion of organizations such as the Society for Women in Radiation Oncology (SWRO). However, this work did not highlight the experiences of female residents in medical physics.

Female representation in medical physics has failed to increase in recent years, as only 21% of those graduating with a bachelor's degree in physics are women. This figure remained unchanged from 2009 to 2019 [3]. This contributes to the fact that physics has the lowest percentage of women obtaining doctoral degrees, with only 19% from 2013 to 2017 [4]. In 2015, a report from the International Organization of Medical Physics (IOMP) found that only 28.9% of medical physicists worldwide were women [5]. An additional study in 2018 found that only 23% of medical physicists in the US were women [6]. By comparison, only 28% of RO physicians were women [1]. Efforts to diversify the field of medical physics are critical to ensure access to this rewarding career for historically underrepresented groups.

Building a family is one factor that can impact the career development trajectory of women in any field, and medical physics is no exception [7]. A qualitative study of medical physicists on work-life balance found that men and women both agreed that women carried the majority of the physical and mental workload at home [7]. While participants of both genders expressed concerns about having a family impacting their opportunities for career advancement, women's concerns about work-life balance particularly influenced their expectations regarding the kind of career they could pursue. Some issues that women respondents highlighted included the impact of motherhood on pursuing an academic career and leadership, struggling to find the flexibility to pursue professional development opportunities, and the burden of childcare. Ways to address work-life conflicts proposed by participants included outsourcing domestic tasks (which is not accessible to all medical physicists), support from colleagues, and childcare availability at the annual American Association of Physicists in Medicine (AAPM) Meeting.

Challenges related to family building, fertility, and work-life integration have been extensively studied in the physician workforce, including among RO physicians. Among female physicians, one in four are diagnosed with infertility [8]. The etiology of the high rate of infertility in the physician workforce is multifactorial. In a sample of medical students through practicing physicians, almost two-thirds (60.1%) delayed childbearing due to medical training [9]. This was not a decision made without consequences; the majority of those who delayed childbearing (55.8%) regretted their decision. The most common reason participants reported (42.0%) for delaying childbearing until after training was that residency required too many hours at work, making parenting after clinical duties challenging. This is consistent with data from RO physicians specifically, where 54% of participants reported that their reproductive plans were limited during residency [2]. In a sample of women oncologists across multiple disciplines, almost all (94.7%) reported that their career plans were at least somewhat associated with the timing of when they started a family, and almost one-fourth (24.2%) said family planning was associated with their decision to choose between academic and private practice [10]. Currently, there is a lack of data on infertility and family building during medical physics training. Medical physicists are in residency training at a similar age to resident physicians; moreover, they likely face many of the same challenges associated with the overlap between prime reproductive years and training.

To continue promoting the success of women in medical physics, collecting more data on gender equity in the profession, including issues related to fertility and family-building challenges, is essential. In 2023, a new survey was distributed to all members of SWRO to assess their experiences five years after the founding of SWRO [11]. An analysis of the responses from medical physicists has yet to be reported. This report presents a sub-analysis of the responses from medical physicists involved in SWRO, highlighting the gender equity issues specifically faced by women and gender minorities in medical physics. Although one transgender participant completed the survey, the limited representation of gender minority participants precluded meaningful conclusions beyond the experiences of women. The findings are intended to inform future research, mentorship initiatives, and institutional efforts aimed at improving equity, inclusion, and professional development within medical physics.

## Materials and methods

Study design and participant eligibility

This study represents a sub-analysis of responses from physicists in a broader cross-sectional survey distributed to members of SWRO. As detailed in the manuscript reporting physician participant outcomes, all current SWRO members, including both physicians and medical physicists, were eligible to participate in the survey [11]. Membership in SWRO is open to all individuals who support the mission of the organization, including men and non-binary individuals. The present analysis focuses specifically on the responses of physicist participants, including both practicing medical physicists and medical physics residents.

Questionnaire development and survey content

An anonymous, 82-question survey was adapted from the original 2016 SWRO pilot founding survey and refined through iterative feedback from a cohort of SWRO leaders [11]. The 2016 pilot founding survey was an internal project conducted among members of the SWRO. Multiple members of the team that conducted the original 2016 SWRO pilot founding survey participated in this newer iteration of the survey. Survey domains included demographics, job satisfaction, mentorship, SWRO membership satisfaction, fertility preservation and family planning, well-being, burnout, productivity, and experiences with gender bias and harassment. Participants were additionally asked to identify perceived barriers to advancement and areas for improvement within SWRO and the field of RO. Both structured survey responses and optional fill-in-the-blank responses were collected to allow participants to further contextualize their experiences. The full survey instrument is included in the Appendix.

Survey administration 

This study was approved by an institutional review board prior to distribution of the survey. Survey administration occurred from January 2023 through February 2023. The survey was distributed electronically to all current SWRO members via the organization's email listserv. In addition, the study was promoted on SWRO social media platforms, including Instagram and Twitter, although survey links were not distributed directly through social media posts.

Qualtrics was used as the data collection platform. Participation was voluntary, and all responses were collected anonymously. No compensation was provided for survey completion. At the time of study recruitment, 26 medical physicists were confirmed SWRO members and were eligible to participate in the survey.

Study variables and outcomes

Survey questions evaluated multiple domains related to the experiences of women and gender minorities in medical physics within RO. Demographic variables included age, gender identity, race and ethnicity, relationship status, parental status, practice setting, country of practice, and training level or professional role.

Additional survey domains assessed perceptions of mentorship, academic productivity, burnout, work-life balance, family planning and fertility preservation, institutional support, satisfaction with SWRO membership, and experiences with gender bias and harassment. Burnout symptoms were assessed using a single self-reported survey item in which participants selected the response that best described their current level of burnout: no symptoms of burnout; occasionally under stress but not burned out; one or more symptoms of burnout; persistent symptoms of burnout; or completely burned out. Participants were also asked about perceived barriers to professional advancement, including limitations in mentorship or funding, clinical workload, and access to childcare.

The survey also evaluated perceptions of family-friendliness within RO, reproductive timing during training, comfort discussing parenthood planning with mentors, and the availability of institutional childcare support. Experiences with unwanted sexual comments, attention, or advances, as well as barriers to reporting harassment, were also assessed.

Analysis

Descriptive statistics were utilized to summarize survey responses. Categorical variables were summarized using frequencies and percentages. Continuous variables, including mean weekly academic time, were summarized descriptively. Fill-in-the-blank responses were qualitatively reviewed and incorporated to contextualize key findings and identify recurring themes raised by participants.

Given the exploratory and descriptive nature of the study and the relatively small sample size of physicist respondents, formal comparative statistical testing was not performed. Findings were qualitatively contextualized against the previously published SWRO physician survey, where relevant [2].

## Results

Participant demographics

The complete characteristics of the physicist survey respondents are presented in Table 1. Of the 26 eligible medical physicist members of SWRO at the time of study recruitment, 20 participated in this study. The majority of respondents were in the 31-35-year age group (n=7, 35%), followed by those aged 26-30 years (n=6, 30%). All identified as women; the vast majority (95%, n=19) were cisgender, and 5% (n=1) were transgender. Participants identified predominantly as White or Caucasian (n=9, 45%), followed by Black or African American (n=6, 30%), Middle Eastern or North African (n=2, 10%), Asian (n=1, 5%), Hispanic/Latino/a/x (n=1, 5%), and other ethnicities (n=1, 5%). Most respondents were married or in a domestic partnership (n=11, 55%), with 35% (n=7) being single and 10% (n=2) being in a relationship. A majority of respondents (n=12, 60%) reported having no children, while 25% (n=5) had one child, and the remaining 15% (n=3) had two or more children. Seventy-five percent (n=15) of participants were practicing medical physicists, and 25% (n=5) were medical physics residents. The majority practiced in the United States (n=14, 70%), with others located in Canada (n=1, 5%) and various other countries (n=5, 25%), including Ghana and the Bahamas. The most common employment setting was an academic or university system (n=14, 70%), followed by government or public-sector roles (n=3, 15%) and private practice (n=2, 10%).

**Table 1:** Participant demographics

Characteristic		N=x (%)
Age		
26-30		6 (30%)
31-35		7 (35%)
36-40		2 (10%)
46-50		3 (15%)
41-45		1 (5%)
>50		1 (5%)
Gender		
Identifying as female		19 (95%)
Identifying as transgender female		1 (5%)
Race/ethnicity		
White or Caucasian population		9 (45%)
Black or African American population		6 (30%)
Middle Eastern or North African population		2 (10%)
Hispanic, Latino or Spanish population		1 (5%)
Asian population		1 (5%)
Other population		1 (5%)
Marital status		
Married or domestic partnership		11 (55%)
Single		7 (35%)
In a relationship		2 (10%)
Parental status		
No children		12 (60%)
1 child		5 (25%)
2 children		2 (10%)
3 children		0
4+ children		1 (5%)
Current position		
Medical physicist		15 (75%)
Medical physicist resident		5 (25%)
Country of practice		
United States		14 (70%)
Canada		1 (5%)
Other		
Ghana		4 (20%)
Bahamas		1 (5%)
Employment setting		
Academic or University system		14 (70%)
Government or public sector		3 (15%)
Private practice		2 (10%)
Resident/fellow/student		1 (5%)

Society for Women in Radiation Oncology membership

When asked about satisfaction with SWRO membership, 60% (n=12) of physicist participants reported being extremely or somewhat satisfied. Only 5% (n=1) of participants reported being extremely dissatisfied, while the remaining participants were neutral (n=7, 35%). Regarding the perceived value of SWRO membership, 65% (n=13) of respondents strongly or somewhat agreed that SWRO has been a good and valuable use of their time. The top reasons for joining SWRO included networking opportunities, advocacy, professional development, mentorship, and increasing the visibility of women in RO. Survey participants also cited similar reasons for maintaining their SWRO membership, with the top reasons being increasing the visibility of women in RO, networking opportunities, and professional development. Most participants (70%, n=14) stated that they were likely or very likely to recommend SWRO membership to a colleague or peer, and 20% (n=4) had already done so. The majority of participants (74%, n=14) also reported satisfaction with specific events offered through SWRO. When asked whether there was a service, activity, or event they would like SWRO to offer, some participants suggested more events specifically related to medical physics, as well as events that increase the representation of medical physicists.

Mentorship

Another subsection of the survey collected information on perceptions of the importance of mentorship in the field of RO (Table 2). Half of the participants reported that having a mentor of the same gender during residency or early in their career was important, and 54% reported that having a mentor in the same program or at the same institution was important. Despite this, only 28% (n=5) were satisfied with the mentorship at their institution, while 44% (n=8) were extremely or somewhat dissatisfied with the mentorship available. Sixty-seven percent (n=12) reported having sought female mentorship outside their own institution. Forty-one percent of participants found mentorship through a personal connection, and 34% found mentorship independently.

**Table 2:** Perceptions of mentorship in RO RO: radiation oncology

Characteristic	N=x (%)
Satisfaction with mentorship in the field	
Extremely satisfied	2 (10%)
Somewhat satisfied	10 (50%)
Neutral	7 (35%)
Somewhat dissatisfied	0
Extremely dissatisfied	1 (5%)
Mentorship as a valuable use of time	
Strongly agree	2 (10%)
Agree	11 (55%)
Neutral	7 (35%)
Disagree	0
Strongly disagree	0
Satisfaction with mentorship​​​​​​​ at institution	
Extremely satisfied	1 (6%)
Somewhat satisfied	4 (22%)
Neutral	5 (28%)
Somewhat dissatisfied	4 (22%)
Extremely dissatisfied	4 (22%)
Sought mentorship​​​​​​​ outside of institution	
Yes	12 (67%)
No	6 (33%)
Reported life balance	
Never	2 (11%)
Rarely	4 (22%)
Occasionally	11 (61%)
Frequently	1 (6%)
Always	0
Reported level of burnout	
No symptoms of burnout	2 (11%)
Occasionally under stress, but not burned out	7 (39%)
One or more symptoms of burnout	6 (33%)
Persistent symptoms of burnout	1 (6%)
Completely burned out	2 (11%)
Protected academic time during the week	
Never	6 (33%)
Rarely	6 (33%)
Sometimes	3 (17%)
Often	1 (6%)
Always	2 (11%)
Reproductive timing impacted by medical training	
Never	6 (33%)
Rarely	6 (33%)
Sometimes	3 (17%)
Often	1 (6%)
Always	2 (11%)
Current or future children affect potential career options	
Strongly disagree	2 (12%)
Somewhat disagree	6 (12%)
Neutral	3 (18%)
Somewhat agree	5 (29%)
Strongly agree	5 (29%)
Gender-specific biases or obstacles	
Strongly disagree	3 (17%)
Somewhat disagree	3 (17%)
Neutral	5 (28%)
Somewhat agree	4 (22%)
Strongly agree	3 (17%)
Encountered unwanted sexual comments, attention, or advances	
Strongly disagree	6 (33%)
Somewhat disagree	2 (11%)
Neutral	2 (11%)
Somewhat agree	6 (33%)
Strongly agree	2 (11%)
Prefer not to answer	0
Refrained from reporting harassment	
Yes	9 (50%)
No	5 (28%)
Prefer not to answer	0
Not applicable	4 (22%)

Well-being, job satisfaction, and burnout

The survey also evaluated levels of well-being, job satisfaction, and burnout among female medical physicists. Five percent (n=1) of participants reported frequently achieving an adequate balance between work, family, relationships, play, and rest. Sixty-one percent (n=11) of participants reported occasionally achieving balance across these domains, and 33% (n=6) reported rarely or never experiencing this balance. Fifty percent (n=9) of participants reported experiencing feelings of burnout.

Productivity and obstacles to advancement

Participants were asked about having protected academic time, defined as hours allotted for research, catch-up, or administrative tasks. Thirty-three percent (n=6) of participants reported sometimes, often, or always having protected academic time. The mean weekly protected academic time was eight hours. Clinical responsibilities (34%), insufficient mentorship (26%), and insufficient funding (16%) were reported as the most common limitations to academic and professional productivity. Limited time and inefficient workflows were also identified as contributing factors.

Fertility preservation and family planning

A significant focus of this survey was to gather information on medical physicists' perceptions of family planning and fertility preservation. Seventy-one percent (n=12) of participants believed that RO is a family-friendly specialty, while 41% (n=7) reported that they experienced RO as family-friendly. Regarding fertility preservation, 85% (n=17) of participants reported that neither they nor their partner/spouse had considered elective fertility preservation, such as oocyte, sperm, or embryo banking. Twelve percent (n=3) of participants had plans to pursue fertility preservation. Twenty-three percent (n=6) of participants reported that they or their partner/spouse had considered, but not pursued, fertility preservation. These participants reported that being unaware of available options (7%), difficulty accessing fertility services (13%), financial constraints (33%), and no longer being interested (40%) were the main reasons they ultimately did not proceed. No survey participants had previously undergone fertility preservation. Forty percent (n=8) of participants reported that their health insurance plan or policy did not offer full or partial coverage for elective fertility preservation, and 55% (n=11) were unsure. One participant reported that their health insurance plan or policy did offer coverage. No participants had ever received information from their program regarding fertility preservation options.

In the context of family planning, 59% of participants believed that their current or future children affected their potential career options, and yet, 41% (n=7) reported being somewhat or extremely dissatisfied with delaying parenthood because of their training and career. Sixty-five percent (n=13) strongly or somewhat agreed that their reproductive timing was impacted by medical training, and 20% (n=11) reported that fear of implications for their career played a role in the timing of childbearing. Thirty percent (n=6) of participants were extremely concerned about their current or future fertility, and an additional 35% (n=7) were somewhat, very, or extremely concerned. One participant became pregnant during residency and had not yet given birth at the time of the survey. This participant reported feeling well or somewhat supported by program directors and administrators. Most participants (70%, n=14) did not feel comfortable discussing parenthood planning with a mentor.

Seven participants had children at the time of completing the survey. Among these participants, 47% reported not having access to childcare at their institution, and 47% reported being unsure whether childcare was available. A small percentage of participants (6%) reported that childcare was available at their institution. Participants reported that their spouse or significant other (43%), parents (14%), or services such as daycare (14%) helped with childcare while they were at work. However, when school issues, doctor appointments, or illnesses arose during work hours, many participants (43%) reported assuming responsibility. A smaller percentage of participants reported that their partner would handle these issues or that responsibility was shared (29% and 29%, respectively). Required evening or early-morning meetings made it difficult to arrange childcare, according to 57% (n=4) of these participants.

Gender bias and harassment

The final goal of this survey was to assess SWRO members' experiences of gender bias and harassment within RO. Thirty-five percent (n=6) of participants either strongly or somewhat agreed that there are gender-specific biases or obstacles for residents or faculty at their respective programs, while 29% (n=5) were neutral. Sixty-one percent (n=11) of participants disagreed that they had increased opportunities for professional advancement because they were female. Regarding sexual harassment, 44% (n=8) of participants reported having encountered unwanted sexual comments, attention, or advances from a superior or colleague. Fifty percent (n=9) of participants reported that they had refrained from honestly reporting harassment on anonymous surveys at their institution because they feared being easily identified. Concerns regarding the feasibility of reporting were cited as a barrier to addressing harassment.

## Discussion

SWRO was originally established in 2017 by women physicians in RO in the United States and Canada, following a survey of women residents that demonstrated a need for a women's RO professional society. Physicists did not participate in the original survey. The primary purpose of this survey-based study was to report changes in the experiences of SWRO physician members over the past five years and to present the perspectives of medical physicists for the first time. In this sub-analysis of the physicist responses, we uniquely highlight the experiences of women and gender minorities within the field of medical physics and identify potential areas for improvement. Although one transgender participant completed the survey, the small number of gender minority respondents limited our ability to draw conclusions regarding gender minority experiences beyond those of women.

Since its inception, SWRO has grown to over 600 members, including 38 medical physicists in 2026. The organization's structure now includes consistent representation of medical physicists on the SWRO Executive Committee. Specifically, the Physics Advocacy Committee was established to welcome physicists into SWRO by promoting inclusivity, organizing programming, and promoting collaboration between RO physicians and medical physicists.

Many scholars have identified a lack of mentorship as a factor affecting career ambitions, including specifically within RO [12]. SWRO established a mentorship program early in its history, pairing interested members to form formal mentorship relationships. This survey reiterated the perceived importance of mentorship within medical physics, with 90% of participants reporting that same-institution mentorship was important and 50% reporting that same-gender mentorship was important. Unfortunately, this report also highlights a lack of satisfactory same-institution mentorship, with only 28% of participants reporting satisfaction in this domain. These findings highlight the importance of national organizations such as SWRO in providing mentorship opportunities that may not exist at individual institutions. Of note, participants were not asked whether they had found mentorship specifically through SWRO; this represents an area for further investigation.

The top three limitations to academic productivity were clinical responsibilities (34%), insufficient mentorship (26%), and insufficient funding (16%). Fifty percent of participants reported that they had refrained from honestly reporting harassment on anonymous surveys at their institution because they feared being easily identified. This is a challenge that will require creative solutions, given the small size of medical physics departments, particularly those with a limited number of women. However, partnering with institutional representatives who have expertise in reporting would be beneficial for improving reporting mechanisms. Free-text responses from this sample highlighted that gender minorities observed opportunities being disproportionately offered to male colleagues and experienced being labeled as "difficult" or "problematic," not feeling welcomed within their institution, and lacking avenues to report negative experiences. Reflecting on departmental practices is necessary to promote change in this area, as a lack of opportunity could further contribute to the disparities in research productivity observed among female radiation physics faculty [13]. Departments should also consider participating in implicit bias training and conducting ongoing evaluations of the distribution of opportunities [14]. Another consideration would be to extend mentorship and professional development opportunities to physicists in community practice.

Through its mentorship programs, professional development opportunities, and advocacy efforts, SWRO has made a positive contribution to networking and the visibility of women in the field. One particularly successful aspect of the organization has been its mentorship program [15]. Among surveyed physicists, 65% found membership valuable, citing networking and mentorship as key benefits. The present survey did not specifically ask participants whether they established mentorship relationships through SWRO. Future studies should evaluate which mentorship initiatives are most beneficial and whether participation in organizations such as SWRO improves access to mentorship over time.

However, the survey also highlighted areas where gender minority physicists continue to face challenges, particularly regarding institutional support and professional advancement. Key obstacles reported included limited access to mentorship, lack of institutional childcare, and gender-specific biases, with 44% of respondents having encountered gender-based harassment and nearly 50% hesitant to report these experiences because of concerns about being identified. In particular, these findings are consistent with those observed in a sub-analysis of physicians [11]. Additionally, challenges in balancing work and family responsibilities remain significant: only 5% of participants reported regularly achieving work-life balance, while the majority identified clinical responsibilities and limited mentorship as factors affecting productivity and career progression. This is consistent with previous data highlighting challenges with work-life balance among women in medical physics [7].

To address these issues, future work should focus on implementing enhanced mentorship programs that provide targeted support within institutions while expanding opportunities for cross-institutional and gender-aligned mentorship. These findings support continued structural efforts to improve mentorship, institutional support, transparency in promotion, and equitable access to career development opportunities for women in medical physics. Offering childcare support within institutions should also be considered, given that over 47% reported a lack of accessible childcare and 57% struggled to accommodate early- and late-work meetings. Early- and late-work meetings pose a significant burden. A previous study of oncology physicians attending multidisciplinary tumor boards highlighted that being a woman or having children was associated with a greater burden from these meetings because of their impact on childcare responsibilities and family dynamics [16]. While medical physicists are unlikely to attend tumor boards, early and late work hours associated with other integral aspects of practice, such as early-morning brachytherapy procedures and late-evening quality assurance activities, may impose a similar burden. Measures such as offering remote options for meetings may foster inclusivity and increase participation.

The data demonstrate a clear need to focus on mentorship and career development for women in medical physics. A deliberate emphasis on career development resources, including webinars and workshops on practical topics such as contract negotiation, billing, coding, and collaborative research, represents one mechanism for action. Specifically, SWRO may collaborate with organizations such as AAPM to develop comprehensive career development resources that are inclusive of medical physicists, further promoting integration and community. Ultimately, these efforts are critical not only for recruiting more women into the field of medical physics but also for retaining them. A longitudinal analysis of women members of AAPM noted that women become full members of the organization, on average, at a younger age than men (34.6 vs 37.5 years); however, women had a higher probability of membership cessation at every age compared with men [17]. In particular, women experience a spike in membership cessation at age 40. Moreover, this contributes to the continued underrepresentation of women in medical physics more broadly, as women represented 24.5% of the full members of AAPM in 2023, compared with 23.5% in 1993 [17].

While SWRO has positively impacted gender equity and support within RO, this survey underscores the need for further structural changes. Enhanced mentorship, greater access to childcare, and continued advocacy against gender-based bias will help address these remaining gaps, ensuring that SWRO remains a vital resource for promoting gender equity and professional development. Future studies should evaluate whether these interventions lead to measurable improvements in recruitment, retention, mentorship, and career advancement.

Limitations of this work include the use of self-reported data and potential selection bias, as participants were recruited exclusively through SWRO membership. Individuals who choose to join organizations such as SWRO may differ from the broader medical physics workforce, including potentially having greater interest in mentorship, advocacy, or issues related to gender equity. Future studies involving broader recruitment through organizations such as AAPM, American Society for Radiation Oncology (ASTRO), American College of Radiation Oncology (ACRO), and other professional societies would improve generalizability. Additionally, the sample size was small, although the response rate of 77% among eligible SWRO medical physics members was high. While recognizing the limitations of the sample size, it is important to consider these data given the limited understanding of women's experiences in medical physics and the relatively small number of women in the field overall. Although the sample was small, it reflects the relatively limited number of women currently practicing within the specialty and provides preliminary insights that warrant larger, multi-organizational investigations. Lastly, despite the study objective of including women and gender minorities, only one transgender participant completed the survey. Therefore, the findings primarily reflect the experiences of women medical physicists and should not be generalized to gender minority populations.

## Conclusions

In summary, this sub-analysis of a recent SWRO survey reports on the experiences of women medical physicists in RO with regard to mentorship, well-being, career development, family planning, and gender bias and harassment. Since SWRO was established, there has been an increase in the representation of medical physicists within the organization. However, challenges for women and gender minorities in medical physics persist, underscoring the need for continued efforts in mentorship, addressing gender-based barriers, and promoting diversity in leadership roles. SWRO's role in fostering a supportive community is highlighted, emphasizing the ongoing need for organizations that promote gender equity in RO.

## Appendices

**Table 3:** SWRO annual survey questions SWRO: Society for Women in Radiation Oncology; ACRO: American College of Radiation Oncology; PGY: postgraduate year; CME: continuing medical education; BF: breastfeeding

SWRO annual survey questions
Demographics
What is your age?
<25
26-30
31-35
36-40
41-45
46-50
>50
With which gender do you most identify?
Female
Male
Transgender female
Transgender male
Non-binary or gender diverse
Prefer not to disclose
Other, please specify
How would you describe yourself? (Select all that apply)
American Indian or Alaskan Native
Asian
Black or African American
Hispanic, Latino/a/x, or Spanish
Middle Eastern and North African
Native Hawaiian or Other Pacific Islander
White or Caucasian
Prefer not to disclose
Other, please specify:
What is your current marital status?
Single
Married/domestic partnership
Widowed
Separated
In a relationship
Other, please specify:
Parental status
No children
1
2
3
4+
What is your current profession or position?
Radiation oncologist
Radiation oncology resident
Medical physicist
Medical physics resident
Medical student
Other, please specify
What is your PGY level? (if resident selected above)
PGY1
PGY2
PGY3
PGY4
PGY5
Which country is your primary practice/work setting located in?
Drop down
How would you best describe your primary employer?
Private practice/community-based system
Government/public sector
Independent contractor/locum tenens
Academic/university system
Industry
Resident/fellow/student
Membership
How satisfied are you with your SWRO membership?
Very dissatisfied
Dissatisfied
Neutral
Satisfied
Very satisfied
Participation in SWRO is a good use of my time.
Strongly disagree
Disagree
Neither agree nor disagree
Agree
Strongly agree
Rank the following in order of importance (1 being most important, 11 being least important) for reasons you joined and became a member of SWRO:
Networking opportunities
Professional development
Mentorship
Advocacy
Increasing visibility of women in radiation oncology
Leadership development
Promotion of diversity within the field (including medical student outreach)
Providing resources for job-hunting and negotiation
Focus on women’s oncologic issues
Political involvement
Other, please specify
Rank the following in order of importance (1 being most important, 11 being least important) for reasons you continue to be a member of SWRO:
Networking opportunities
Professional development
Mentorship
Advocacy
Increasing visibility of women in Radiation Oncology
Leadership development
Promotion of diversity within the field (including medical student outreach)
Providing resources for job-hunting and negotiation
Focus on women’s oncologic issues
Political involvement
Other, please specify
How do you stay informed about SWRO activities and announcements? Select all that apply.
By opening emails sent by SWRO
Reading SWRO blog
Visiting the SWRO website
Follow SWRO/SWRO members on Facebook
Follow SWRO/SWRO members on Twitter
Other, please specify
Not all members of SWRO have Twitter/Facebook Accounts. We are trying to get a feel for interest in creating an Instagram account. Are you currently an Instagram User?
Yes, regularly use
Yes, but rarely use
No, do not have an account
If we created an Instagram, would you follow for staying up to date on upcoming ways to get involved & events?
Yes
No
Unsure
How likely is it that you would recommend membership in SWRO to a colleague or peer?
Unlikely
Neither likely nor unlikely
Likely
Very likely
How would you rate your overall satisfaction with SWRO's educational/professional development offerings (e.g, live virtual meetings, on-demand meetings, CME, etc.)?
Very dissatisfied
Somewhat dissatisfied
Neutral
Somewhat satisfied
Very satisfied
Is there a service, activity, or event you wish SWRO offered that it does not at present?
Fill in the blank
If SWRO offered occasional in-person events at local restaurants in major cities in the US, would you be interested in participating and meeting other SWRO members at such events?
Yes
No
Depends, please explain:
About you and your career job/position satisfaction
How satisfied are you with your current job?
Very dissatisfied
Dissatisfied
Neutral
Satisfied
Very satisfied
Not applicable
Prefer not to answer
Mentorship
How satisfied are you with the current mentorship available to you at your institution?
Very dissatisfied
Dissatisfied
Neutral
Satisfied
Very satisfied
Do you have mentors from the following categories? (select all that apply)
Female?
Male?
Resident?
Fellow?
Attending physician?
At your “home” program?
At a different program?
Self-Introduction?
Professional society?
How satisfied are you with the current female mentorship available to you at your institution?
Very dissatisfied
Dissatisfied
Neutral
Satisfied
Very satisfied
Have you/do you seek female mentorship outside of your own institution?
Never
Rarely
Sometimes
Often
Always
How satisfied are you with the current female mentorship available to you within the radiation oncology field?
Very dissatisfied
Dissatisfied
Neutral
Satisfied
Very satisfied
How have you previously connected with female radiation oncology mentors? (select all that apply)
“Home program” for medical school
“Home program” for residency
Away Rotations from medical and/or residency
Personal connection
SWRO’s mentorship program
Other formal mentorship programs (e.g., ACRO mentorship program, NextGen Brachy)
Found my own
Other, please specify:
Productivity
Do you have protected academic time during the week?
Never
Rarely
Sometimes
Often
Always
Not applicable
On average, how many hours per week are allotted to you for research time specifically? (If applicable)
Enter number
What do you feel are the top 3 limitations to your professional productivity? Please rank in order.
I do not feel there are limitations to my productivity
Insufficient funding
Insufficient mentorship
Clinical responsibilities
Studying
Child care
Housework/errands
Significant other
Other family members or friends
Other, please specify:
Not applicable
Obstacles
To what extent do you agree with the following statement? ”There are gender-specific biases or obstacles for residents in my program. ”
Strongly agree
Agree
Neutral
Disagree
Strongly disagree
Prefer not to answer
To what extent do you agree with the following statement? “I have felt denied or excluded from opportunities for professional advancement due to my gender?”
Strongly agree
Agree
Neutral
Disagree
Strongly disagree
Prefer not to answer
To what extent do you agree with the following statement? “I feel I have had increased opportunities for professional advancement due to my gender?”
Strongly agree
Agree
Neutral
Disagree
Strongly disagree
Prefer not to answer
Harassment
To what extent do you agree with the following statement? “I have encountered unwanted sexual comments, attention or advances by a superior or colleague?”
Strongly agree
Agree
Neutral
Disagree
Strongly disagree
Prefer not to answer
Have you refrained from honestly reporting harassment on anonymous surveys at your institution due to fear of being easily identified?
Yes
No
Prefer not to answer
Well-being
For the next few sections, we would like to better understand your wellbeing.
Overall, based on your definition of burnout, how would you rate your level of burnout?
I enjoy my work. I have no symptoms of burnout.
Occasionally I am under stress, and I don't always have as much energy as I once did, but I don't feel burned out.
I am definitely burning out and have one or more symptoms of burnout, such as physical and emotional exhaustion.
The symptoms of burnout that I'm experiencing won't go away. I think about frustration at work a lot.
I feel completely burned out and often wonder if I can go on. I am at the point where I may need some changes or may need to seek some sort of help.
Over the past academic year, how often did you feel that you achieved an adequate balance between work, family, relationships, play and rest?
Never
Rarely
Occasionally
Frequently
Always
Maternity, family, and medical leave
Family
To what extent do you agree with the following statement? “Radiation Oncology as a field is perceived as family friendly”
Strongly agree
Agree
Neutral
Disagree
Strongly disagree
Prefer not to answer
To what extent do you agree with the following statement? “Radiation Oncology as a field is actually “family friendly””
Strongly agree
Agree
Neutral
Disagree
Strongly disagree
Prefer not to answer
To what extent do you agree with the following statement? “My current and/or potential future children affect my potential career options”
Strongly agree
Agree
Neutral
Disagree
Strongly disagree
Prefer not to answer
If you have children, who primarily cares for them while you are at work?
Spouse/significant other
Your parent(s)
Your spouse’s or significant other’s parent(s)
Other family member
Daycare
Nursery school
Nanny/babysitter
Does not require childcare
Other, please specify:
If you have children, who takes care of any school issues/doctor appointments/illnesses that arise?
Myself
My partner
Other family member
A nanny
Shared responsibility
Does your place of work have available childcare?
Yes
No
I am not sure
To what extent do you agree with the following statement? “Required evening or early morning meetings make it difficult to arrange child care.”
Strongly agree
Agree
Neutral
Disagree
Strongly disagree
Parenthood planning
To what extent do you agree with the following statement? “My reproductive plans have been impacted by my medical training.”
Strongly agree
Agree
Neutral
Disagree
Strongly disagree
Not applicable
Prefer not to answer
What factors contribute(d) to the impacts on your childcare plans in training?
Stated pressure from program leadership
Unstated pressure from program leadership
Stated pressure from coresidents
Unstated pressure from coresidents
Desire not to extend total training time
Fear of implications on career
Fear of appearing undesirable in the job market with new children or pregnant during interviews
Desire not to place undue “burden” on colleagues
Limitation on available leave time
Physical/health limitation
Financial limitation
Childcare limitation
Abortion laws in my state of residence
Other: please specify
Prefer not to answer
If you have delayed parenthood, how satisfied are you with the decision?
Very satisfied
Satisfied
Neither
Dissatisfied
Very dissatisfied
Not applicable
Do you have a mentor you would feel comfortable discussing parenthood planning with if you desired to?
Yes
No
Pregnancy during residency
How many times have you or your partner become pregnant during your resident program?
0
1
2
3
4+
Not applicable
If you have been pregnant during residency, during what year of residency have you/will you deliver? (select all that apply)
After residency
PGY1
PGY2
PGY3
PGY4
PGY5
PGY6
I have not been pregnant during residency
Support- during your/your partner’s most recent pregnancy, how emotionally supported did you feel by…
Your co-residents
Not supported
Somewhat not supported
Neutral
Somewhat supported
Well-supported
Your attendings
Not supported
Somewhat not supported
Neutral
Somewhat supported
Well-supported
Your program director and administration
Not supported
Somewhat not supported
Neutral
Somewhat supported
Well-supported
Support- During your most recent pregnancy, how logistically supported did you feel by…
Your co-residents
Not supported
Somewhat not supported
Neutral
Somewhat supported
Well-supported
Your attendings
Not supported
Somewhat not supported
Neutral
Somewhat supported
Well-supported
Your program director and administration
Not supported
Somewhat not supported
Neutral
Somewhat supported
Well-supported
Parental- Please answer the following for your most recent child’s birth
Leading up to your most recent child’s birth, approximately how many weeks of parental leave did you take?
Numeric text: ______ weeks
After your most recent child’s birth, approximately how many weeks of parental leave did/will you take?
Numeric text:______ weeks
After your most recent child’s birth, approximately how many weeks of parental leave did/will your partner take?
Numeric text:______ weeks
Please choose up to 3 factors that most determined the length of your parental leave.
Ability to obtain child care
Desire to not delay taking my board exam
Desire to not extend my residency training further
Finances
Fellowship or job start date restrictions
Infant health complications
Maternal health complications
Newborn bonding
Partner’s parental leave
Program mandated leave
Repercussions to my colleagues
Repercussions to my relationships with supervision physicians
Other, please specify
By how many weeks will your training be extended by your parental leave? (leave blank if not yet been determined)
Numeric text: ______ weeks
How many weeks of leave were you allowed to take before you would need to extend your residency?
Numeric text:______ weeks
For how many weeks of leave were you paid (excluding sick leave and vacation)?
Numeric text:______ weeks
Approximately what percentage of your salary were you paid?
Slider numbers: 0, 50, 100)
How satisfied are you with the length of your maternity leave?
Less than I would like
About right
More than I would like
When you returned/return to work, how was/will your schedule be adjusted for reentry? (choose all that apply)
No adjustments, returned to full-time work
Part time
Research time
Less inpatient time
Other, please specify
Do you need to "pay back" a call that was/will be missed over your maternity leave?
Yes
No
Took extra call before leave
I am not sure
Breastfeeding
Did you plan to breastfeed your baby?
Yes
No
For approximately how many months did you provide breastmilk for your baby, or have you provided so far?
Numeric text: ______ months
How satisfied are you with the length of time you were able to provide breastmilk for your baby?
Less than I would like
About right
More than I would like
What barriers to breastfeeding did you encounter upon returning to work? (Please select all that apply)
Insufficient time to pump at work
No appropriate place to pump at work
Lack of attending and/or colleague support
Inability to maintain supply
Infant health or lack of infant interest in BF
Other
Please specify: _____
Not Applicable
Overall
What would improve the experience of pregnancy and parenthood in residency?
Free text
Questions for Current Residents (only will show up if they answer yes to being a current resident)
How many total residents are in your program?
Numeric text
How many total female-identifying residents are in your program?
Numeric text
How many total attending physicians are there in your residency program?
Numeric text
How many total attending physicians in your residency program are women?
0
1
2
3
4
5 or more
To what extent do you agree with the following statement? “Females are under-represented in my residency program”
Strongly agree
Agree
Neutral
Disagree
Strongly disagree
Closing questions
Do you have additional thoughts that you would like to share?
Free text
What other benefits and/or services can SWRO provide to assist you in your practice?
Free text
Thank you for taking the time to complete this survey. Your response is important to us and will be used to continue to improve our service to you.
